# A Systematic Review of Beta Cell Function in Adults of Black African Ethnicity

**DOI:** 10.1155/2019/7891359

**Published:** 2019-10-20

**Authors:** M. Ladwa, O. Hakim, S. A. Amiel, L. M. Goff

**Affiliations:** Diabetes Research Group, Department of Diabetes, School of Life Course Sciences, Faculty of Life Sciences & Medicine, King's College London, London, UK

## Abstract

**Background:**

Understanding ethnic differences in beta cell function has important implications for preventative and therapeutic strategies in populations at high risk of type 2 diabetes (T2D). The existing literature, largely drawn from work in children and adolescents, suggests that beta cell function in black African (BA) populations is upregulated when compared to white Europeans (WE).

**Methods:**

A systematic literature search was undertaken in June 2018 to identify comparative studies of beta cell function between adults (>age 18 years) of indigenous/diasporic BA and WE ethnicity. All categories of glucose tolerance and all methodologies of assessing beta cell function *in vivo* were included.

**Results:**

41 studies were identified for inclusion into a qualitative synthesis. The majority were studies in African American populations (*n* = 30) with normal glucose tolerance (NGT)/nondiabetes (*n* = 25), using intravenous glucose stimulation techniques (*n* = 27). There were fewer studies in populations defined as only impaired fasting glucose/impaired glucose tolerance (IFG/IGT) (*n* = 3) or only T2D (*n* = 3). Although BA broadly exhibited greater peripheral insulin responses than WE, the relatively small number of studies which measured C-peptide to differentiate between beta cell insulin secretion and hepatic insulin extraction (*n* = 14) had highly variable findings. In exclusively IGT or T2D cohorts, beta cell insulin secretion was found to be lower in BA compared to WE.

**Conclusions:**

There is inconsistent evidence for upregulated beta cell function in BA adults, and they may in fact exhibit greater deficits in insulin secretory function as glucose intolerance develops.

## 1. Background

Populations of black African (BA) ethnicity have a higher prevalence [1, 2] and earlier age of onset [3, 4] of type 2 diabetes (T2D) compared to those of white European (WE) ethnicity. While there is evidence that part of the disparity is due to environmental and cultural factors (such as socioeconomic status and diet) [5, 6], studies which adjust for these variables have found persistently higher rates of T2D and poorer glycaemic control in BA populations [7, 8], suggesting that ethnic-specific pathophysiological differences also play a role.

It has been hypothesised that in BA populations, beta cell function is upregulated or exaggerated in comparison to WE [9, 10], possibly mediated by lower adiponectin levels [11, 12], greater sensitivity of the beta cell to free fatty acid (FFA) stimulation [13, 14], or dietary factors such as an increased fat-to-carbohydrate ratio [15, 16]. This appears to be borne out by a meta-analysis of ethnic differences in insulin secretion by Kodama et al. [17], which concludes that BA exhibit a higher acute insulin response to glucose (AIRg, as measured by the intravenous glucose tolerance test) compared to WE. It has been speculated that this state of “upregulated” beta cell function plays a role in the increased risk of T2D in BA by predisposing to premature beta cell exhaustion [9].

There is no widely accepted “gold standard” method of assessing beta cell function *in vivo*. The most common techniques measure insulin response following the stimulation of the beta cell by glucose, either intravenously (as in the case of the hyperglycaemic clamp, graded glucose infusion, or intravenous tolerance test, which may be modified by intravenous insulin or tolbutamide) or orally (following the oral glucose tolerance test (OGTT) or the mixed meal tolerance test (MMTT)) [18]. Other techniques use intravenous arginine or glucagon to provoke a robust insulin secretory response [18]. Surrogate indices are also used, which may be derived from fasting glucose and insulin, such as the homeostatic model assessment of beta cell function (HOMA%B), or from the OGTT/MMTT, such as the insulinogenic index or the corrected insulin response (CIR) [18]. Each method has its strengths and limitations; for example, oral glucose and mixed meal tests are highly physiological while intravenous techniques allow specific assessment of the beta cell by excluding the modulating effect of the incretin hormones [18, 19].

There are two important factors to consider when assessing the evidence for “upregulated” beta cell function in BA. Firstly, “beta cell function” implies the concept of the beta cell adequately meeting its physiological role of maintaining glucose homeostasis; that is, it requires assessment of insulin secretion not in isolation but in the context of prevailing insulin sensitivity. Secondly, peripheral insulin levels are determined by both the rate of insulin secretion and the rate of hepatic insulin extraction (HIE), as insulin is secreted by the pancreatic beta cell into the portal vein and undergoes first pass metabolism in the liver before entering the systemic circulation [20]. As C-peptide is cosecreted with insulin into the portal vein in equimolar quantities and undergoes negligible hepatic extraction, measurements of plasma C-peptide are a better reflection of beta cell insulin secretion than plasma insulin levels [21].

The purpose of this systematic review is to examine the evidence for the impact of BA ethnicity on physiological differences in beta cell function in adulthood, taking into account both adjustments made for insulin sensitivity and the differentiation between beta cell insulin secretion and HIE. Unlike previous reviews, it examines adults only, as paediatric populations with impaired glucose regulation are likely to represent a more extreme phenotype. Furthermore, this review will include studies employing a variety of methodologies, in order to obtain a more comprehensive review of ethnic differences in beta cell function.

## 2. Methods and Procedures

### 2.1. Search Strategy

The study was formulated with reference to the Preferred Reporting Items for Systematic Reviews and Meta-Analyses (PRISMA) [22].

A modified “PICO” (Population, Intervention, Comparison, Outcome) framework was used. As the relevant topic is ethnic difference rather than intervention, “Phenomenon of Interest” was substituted for “Intervention” and “Outcome.”

Using this framework, the following question was generated: “how does beta cell function in adults of black African ethnicity differ from that of adults of white European ethnicity across all ranges of glucose tolerance?”

The Ovid Medline database was searched in June 2018 to identify potentially relevant publications. Keywords included “African”, “Caribbean”, “beta cell function”, “acute insulin response (AIR)”, “disposition index (DI)”, “insulinogenic index”, and “insulin secretion”. The complete search string may be found in the appendix. No limits were set in terms of publication date or language.

Inclusion and exclusion criteria were predetermined in order to systematically select studies.

- Inclusion criteria
  - Population: adults (over 18 years of age) of black African ethnicity. To include both indigenous populations and those of the diaspora, e.g., African Americans, African-Caribbean, and indigenous African. Both male and female. Across all ranges of glucose tolerance: normal glucose tolerant, impaired glucose tolerant, impaired fasting glucose, and type 2 diabetic
  - Phenomenon of interest: beta cell function. Indices included were HOMA%B, insulin secretion, AIR (acute insulin response), EIR (early insulin response), insulinogenic index, corrected insulin response (CIR), disposition index (DI), beta cell responsivity to glucose (Phi1, Phi2, and Phi total), insulin secretion rate (ISR), and insulin secretory function (ISF)
  - Comparison population: adults of white European ethnicity. To include whites, Caucasians, non-Hispanic whites, and white Europeans
- Exclusion criteria
  - Population: population contains only subjects < 18 years
  - Phenomenon of interest: no assessment of beta cell function, e.g., only genetic data collected, only insulin clearance assessed, only fasting insulin/C-peptide without model assessment
  - Comparison population: no direct *statistical* comparison to WE population (or data not reported in comparison to WE population)

Two investigators (ML and OH) independently screened the search results to determine study inclusion to minimise bias. In the first step, studies were eliminated if the abstract indicated that at least one criterion was not met. In the second step, full-text manuscripts were obtained from the remaining studies to assess them against the inclusion and exclusion criteria. The references of included studies were also reviewed to identify further suitable studies.

If data from the same study were reported in multiple publications, only the publication with the greatest number of participants in the analyses was included.

### 2.2. Quality Assessment

The Newcastle-Ottawa scale [23] (developed as a quality assessment tool for nonrandomised studies) was used to determine the quality and risk of bias of the selected papers. A modified version of the scale for cross-sectional studies (mNOS) was formulated (see the appendix).

In the absence of formal thresholds for rating quality, studies were judged to be at high risk of bias if they scored 5 stars or below on the mNOS and low risk of bias if they scored 6 stars or above.

## 3. Results

**Figure 1:**
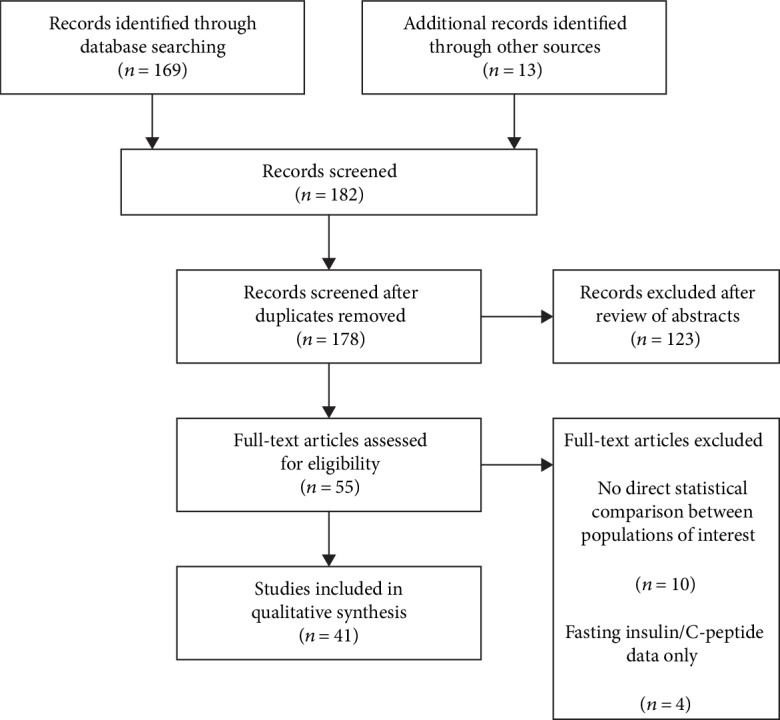
Study selection flow chart. Adapted from PRISMA 2009 [22].

The selection of the included studies is shown in Figure 1. A total of 182 articles were screened; after the study selection process, 41 studies met the prespecified eligibility criteria and were included in the qualitative synthesis.

The characteristics of the included studies are shown in Table 1. The majority were studies of African American populations (*n* = 30), but other study populations included indigenous black African (*n* = 5) [24–28], immigrant black African (*n* = 2) [29, 30], UK African-Caribbean (*n* = 3) [31–33], and a mixture of the above (*n* = 1) [34]. The total number of subjects of BA ethnicity in the included studies was 4619. The smallest cohort of BA subjects was 7 [24] and the largest was 752 [35]. The majority of studies (*n* = 25) were in NGT or nondiabetic populations only; three studies were in prediabetic (IFG/IGT) populations only [35–37], and three studies were in populations with T2D only [30, 32, 38]. Ten studies were in a population known to be of mixed glucose tolerance [31, 39–47].

The studies comprised 17 all-female cohorts [25–28, 37, 41, 43, 48–56], 3 all-male cohorts [24, 29, 30], and 21 mixed-sex cohorts. Where sex of subject was reported by ethnicity, the majority in both BA (3350 of 4395, or 76%) and WE (6630 of 10900, or 61%) subjects were female. There was evidence of sex-specific differences in insulin secretion within the BA population, with females exhibiting a greater insulin response compared to males [33, 57].

A variety of methodologies were employed to assess beta cell function, with some studies employing multiple methods. These included models based on fasting parameters (*n* = 5) [32, 36, 37, 41, 58] and measurements using data from oral glucose and mixed meal stimulation tests (*n* = 21), such as poststimulation insulin and/or C-peptide concentrations [24, 25, 28, 31, 37, 38, 50], corrected insulin response (CIR) [35, 42], insulinogenic index [27, 36, 40, 44, 55], and insulin and/or C-peptide area under the curve (AUC) [26, 29, 30, 34, 42, 48, 59, 60]. Of the studies using intravenous stimulation (*n* = 27), some studies employed multiple methods within the same study, most commonly the insulin-modified IVGTT (*n* = 15) [27, 36–39, 43–45, 49, 51–54, 56, 58]. Studies also employed the nonmodified IVGTT (*n* = 6) [33, 46, 59–62], the tolbutamide-modified IVGTT (*n* = 4) [43, 44, 51, 63], the hyperglycaemic clamp (*n* = 4) [30, 47, 64, 65], and the arginine-stimulated response (*n* = 2) [47, 60]. One study [25] used combined tolbutamide and glucagon intravenous stimulation.

Nineteen studies reported measurements of insulin secretion corrected for insulin sensitivity, with adjustment by HOMA-IR [28, 40], *M* value from hyperinsulinaemic-euglycaemic clamp [59, 62], insulin sensitivity index (ISI) [38, 42, 43], or by calculation of the disposition index (AIR × Si) [27, 33, 36, 37, 44, 45, 47, 53, 54, 58, 60, 61]. According to the prespecified quality criteria, one study was at high risk of bias (*n* = 1) [24] while the remainder were at low risk of bias (*n* = 40).

As the study population sizes are very different, the cumulative *n* has been calculated and presented for Tables 2 and 3; however, due to the high degree of variability in study populations and methodologies, this is intended to be indicative rather than for direct quantitative comparison.

### 3.1. Overall Findings

The majority—thirty-four out of forty-one studies—found evidence of a higher peripheral insulin response in people of BA compared to WE ethnicity.

#### 3.1.1. Adjustment for Adiposity

Some studies (*n* = 14) controlled for measures of adiposity, whether using surrogate measurements such as waist circumference or waist-hip ratio [28, 38, 39, 41, 60], or using hydrostatic weighing [49] or DXA to assess percentage body fat [27, 36, 37, 42, 52, 55, 59], or using CT imaging to assess volume of visceral and subcutaneous fat deposits [54]. All 14 studies consistently demonstrated that hyperinsulinaemia of BA persisted after adjustment for adiposity.

#### 3.1.2. Adjustment for Insulin Sensitivity

The relative hyperinsulinaemia of BA ethnicity persisted in the majority of studies which adjusted for the prevailing insulin sensitivity (*n* = 15), while a minority (*n* = 2) of studies found that hyperinsulinaemia was an appropriate compensatory response to higher insulin resistance [27, 33].

#### 3.1.3. Models Based on Fasting Measures

Five studies calculated HOMA%B using fasting glucose and fasting insulin. Two studies, one in a nondiabetic and one in a mixed NGT/IFG population, found higher HOMA%B in BA [41, 58], one study in an IFG/IGT population found no significant ethnic difference [37], and two studies in IFG/IGT and type 2 diabetic populations found that HOMA%B was lower in BA compared to WE [32, 36].

#### 3.1.4. Insulin Response Post Oral Glucose or Meal

Of the 21 studies reporting indices of insulin secretion based on oral glucose or meal tests (Table 2), the majority found no ethnic difference in insulin response between BA and WE (*n* = 10).

Seven studies found a greater insulin response in BA [34, 35, 42, 44, 48, 50, 55] while two studies found a lower response [24, 25]. Two studies had different results in stratified cohorts, with a greater insulin response in BA in NGT subjects but no ethnic difference in IGT or T2D subjects [40] and a greater insulin response in BA in obese subjects but no ethnic difference in lean subjects [28].

Only three studies [26, 29, 30] employed the mixed meal test, while one study found a higher insulin levels at 30 mins post meal in BA [26]; none of these studies found significant ethnic differences in the incremental area under the curve for insulin post meal.

#### 3.1.5. Insulin Response Post Intravenous Glucose

A consistent picture emerges from the 25 studies which assessed insulin response to intravenous glucose (Table 2), with the overwhelming majority finding that the insulin response was greater in those of BA compared to WE ethnicity, across all categories of glucose tolerance (*n* = 23). One study in a T2D population found no ethnic difference [30], and one study in an NGT population found the response was lower in BA compared to WE [25].

#### 3.1.6. Beta Cell Insulin Secretion Using C-Peptide Measurements

Fourteen out of forty-one studies used C-peptide measurements in their assessment of beta cell function (see Table 3). Most used oral glucose stimulation techniques (*n* = 9), with a minority using intravenous techniques (*n* = 3) [52, 56, 63] or a combination of both oral and intravenous stimulation (*n* = 2) [25, 30]. In NGT cohorts, findings were conflicting, with some studies finding that beta cell insulin secretion was higher in BA vs. WE (*n* = 4) [28, 48, 52, 56] while others found it was lower (*n* = 2) [25, 50] or that there was no significant difference (*n* = 2) [28, 29, 34, 63]. In exclusively IGT or T2D cohorts, beta cell insulin secretion by C-peptide measurement was found to be consistently lower in BA compared to WE, albeit the number of studies was very small (*n* = 3, comprising a total of 170 BA subjects) [30, 36, 37]. In two populations of mixed glucose tolerance, no significant difference was found [31, 42].

## 4. Discussion

### 4.1. Overall Findings

This systematic review is aimed at examining the evidence for “upregulated” beta cell function in adults of black African ethnicity; in particular, it sought to account for prevailing insulin sensitivity and to differentiate between insulin secretion and hepatic insulin extraction.

Overall, the results show that adults of black African ethnicity—whether indigenous or of the diaspora—have a greater peripheral insulin response compared to those of white European ethnicity. Their relative hyperinsulinaemia does not appear to be accounted for either by differences in insulin sensitivity or differences in adiposity.

Hyperinsulinaemia in black African populations appears to be a highly conserved trait [34] which has been demonstrated in prepubertal children [66] and which may be driven by both genetic and epigenetic factors [67]. It has been hypothesised that a robust insulin response may have evolved in this population to promote tissue growth, which is in keeping with the observation that BA youths tend to be taller than their white counterparts [68] and that BA populations have increased bone density [69–71] and muscle mass [72, 73] compared to WE.

However, there are several areas where this systematic review has demonstrated limitations or inconsistencies in our established understanding. There appear to be four key areas of methodology which affect the outcomes of each study: the use of C-peptide measurements to assess insulin secretion; the use of oral or intravenous methods to stimulate beta cell response; the glucose tolerance status of the study population; and, possibly, the sex of the population.

### 4.2. Effect of C-Peptide Measurement

Only 14 out of the 41 studies measured C-peptide responses and were therefore able to differentiate between insulin secretory function and hepatic insulin extraction. This is of great importance, given that the evidence suggests that HIE is significantly lower in black compared to white ethnic populations [56, 66]. While the majority of studies found peripheral hyperinsulinaemia in BA adults, the use of C-peptide levels as a measure of beta cell insulin secretion gave rise to highly variable findings. Although direct quantitative comparison is not possible due to the heterogeneity of populations and methods used, the cumulative *n* presented in Table 3 suggests that the weight of evidence based on C-peptide measurements does not support the finding of beta cell upregulation in BA adults. This is in contrast to the work in children and adolescents, which has found both increased beta cell insulin secretion and reduced HIE in BA [15, 74, 75]. Whether BA adults exhibit increased beta cell secretion or whether their hyperinsulinaemia is driven predominantly by reduced HIE remains an unresolved question.

The differences between the findings in children and adults may be due to an age-related decline in beta cell function [76, 77], with reduced HIE playing a relatively more dominant role in hyperinsulinaemia of BA adults. It is interesting that HIE appears to be an important physiological process underlying ethnic differences in glucose metabolism. It has been previously noted that HIE is the primary cause of hyperinsulinaemia in subjects with more severe glucose intolerance [78] and that it may be an important determinant of future T2D in BA [79]. While reduced HIE is traditionally understood to be associated with visceral adiposity and increased levels of hepatic fat [80, 81], in BA populations there is conversely evidence of lower intrahepatic lipid compared to WE [82–85]. Therefore, the mechanism of reduced HIE in BA is yet to be fully determined, but potential routes of investigation include the role of inflammatory and vascular mediators [86, 87].

### 4.3. Effect of IV versus Oral Methods of Beta Cell Stimulation

Where previous reviews have been drawn from mainly intravenous studies [9, 17], here the inclusion of multiple methodologies of beta cell function assessment gives a more complex picture. Studies examining the response to intravenous glucose administration provide highly consistent evidence for hyperinsulinaemia in BA, whereas studies using oral glucose or meal ingestion have much more variable findings. The discrepancy between intravenous and oral studies has been previously noted in the literature [9] and remains largely unexplained. Differences in the incretin response are one possible mechanism, but there are no consistent findings from the few studies which have investigated ethnic differences in the incretin pathway [42, 88–90].

These observations call into question whether the ethnic differences seen during intravenous studies are clinically relevant if they cannot be reliably demonstrated under physiological conditions. In particular, the small subset of studies using arguably the most physiological method of assessment, i.e., the mixed meal tolerance test, did not find any ethnic differences in insulin response. Further investigation is needed to determine the mechanisms which lead to the route of delivery and the magnitude of the glucose load provoking different insulin responses in BA adults.

### 4.4. Effect of Glucose Tolerance Status

It should be noted that not all studies measured glucose tolerance as part of their protocols; hence, while the population was defined as “healthy” or “nondiabetic” it is conceivable that participants with impaired glucose regulation were included in the sample. Furthermore, many studies were comprised of cohorts of mixed glucose tolerance. Therefore, an attempt to examine ethnic differences by glucose tolerance was limited by the small number of relevant studies.

While there were only three studies which assessed insulin secretory function by C-peptide measurements in IGT/IFG or T2D cohorts where glucose tolerance was strictly defined [30, 36, 37], all three of these indicated that BA adults with impaired glucose tolerance and T2D exhibit greater insulin secretory deficits compared to WE. Interestingly, this is in direct contrast to data from paediatric populations, which demonstrates elevated insulin secretory function in BA across all categories of glucose tolerance [10, 75, 91, 92].

It may be that impaired glucose regulation in the paediatric/youth population represents a more extreme or aggressive phenotype compared to adults. In youth, glucose intolerance is likely to be associated with severe obesity [93] which promotes beta cell hypersecretion of insulin, whereas age-related beta cell decline in BA adults may account for their relatively greater insulin secretory deficits as they progress to T2D. The findings of this review raise the question of whether the beta cells of BA adults are more vulnerable to dysfunction than their WE counterparts as obesity and insulin resistance prevail.

### 4.5. Effect of Gender

Although sex-specific differences were not explored by the majority of the studies, two studies found evidence that BA females exhibit greater hyperinsulinaemia compared with BA males [33, 57]. Enhanced postprandial insulin secretion in females compared to males has also been demonstrated in other ethnic groups, including white Americans [94] and East Asians [95]. While there was a predominance of female subjects in both ethnic groups (61% of WE and 76% of BA subjects across all included studies), the relatively higher proportion of females in the BA cohorts may have led to an overestimation of ethnic differences.

## 5. Conclusions

While BA have a hyperinsulinaemic response to glucose, reduced hepatic insulin extraction rather than differences in beta cell function may be the primary determinant of ethnic differences in diabetes pathophysiology in adulthood. The available literature is predominantly drawn from female, NGT/nondiabetic subjects, and there are relatively few studies which look at exclusively IFG/IGT or T2D populations or which take differences in HIE into account. Furthermore, with the exception of responses to intravenous glucose, the reported direction and magnitude of differences in insulin responses to glucose challenges are not consistent across all studies. The methodology employed—namely, whether intravenous or oral techniques are used, whether C-peptide levels are assessed, and/or whether the glucose tolerance status of the population is studied—appears to have a significant impact on the findings made. The mechanisms of hyperinsulinaemia in BA adults, and how these may relate to their increased risk of T2D, therefore remain unclear.

The cumulative evidence demonstrates that further work is needed to determine these mechanisms, using rigorous methodology to differentiate between insulin secretion and insulin clearance, adjusting for insulin sensitivity, using both oral and intravenous techniques and examining subjects according to strictly defined categories of glucose tolerance.

## Figures and Tables

**Table 1:** Summary of characteristics of the included studies: methods, population of interest, glucose tolerance status, findings, and mNOS.

Study	Methods	Study design	Population of interest					Comparison population		Findings	mNOS
			Ethnicity	*n*	Mean age	Gender %M/F	Glucose tolerance		*n*		
Rubenstein et al. [24]	OGTT	Cross-sectional	Native black South African, no FHx of T2D	7	30 ± 6	100/0	NGT	White South African, no FHx of T2D	8	Similar fasting insulin levels, but lower serum insulin in response to oral glucose and lower renal insulin clearance in BA compared to WE	5
Shires et al. [25]	“Maximal beta cell stimulation”—75 g oral glucose followed by IV tolbutamide and glucagon stimulation	Cross-sectional	Obese native black South African	10	39.4 ± 1.7	0/100	Nondiabetic	Obese white South African	8	Serum insulin and C-peptide levels significantly lower in BA compared to WE 30 mins after oral glucose and 30 mins after tolbutamide and glucagon	6
Cruickshank et al. [31]	OGTT	Cross-sectional	UK Afro-Caribbean	106	56.6 ± 6.0 (F), 57.0 ± 5.0 (M)	50/50	NGT, IGT, and T2D	White European	101	No statistically significant difference between C-peptide and insulin concentrations post oral glucose; however, difference in profiles led investigators to suggest impaired hepatic processing of insulin in BA.	6
Osei et al. [48]	OGTT	Case control	African American (first-degree relatives of T2D and controls)	24 relatives and 8 controls Total = 32	32 ± 2 (relatives) and 27 ± 2 (controls)	0/100	NGT	White American (first-degree relatives of T2D and controls)	22	In both first-degree relatives and controls, iAUC insulin and C-peptide were significantly higher in BA compared to WE.	9
UK Prospective Diabetes Study Group [32]	Fasting glucose and insulin to derive HOMA%B	Prospective cohort	UK Afro-Caribbean	387	51.6 ± 7.4 (M) 50.2 ± 7.2 (F)	57/43	T2D	UK Caucasian	4177	Lower HOMA%B in BA compared to WE, adjusted for age and BMI. BA had higher fasting plasma glucose and higher HbA1c vs. WE.	9
Osei et al. [63]	Tolbutamide-modified IVGTT	Cross-sectional	African American	32	—	0/100	NGT	White American	30	Higher insulin levels in BA but similar C-peptide levels compared to WE	9
Haffner et al. [39]	Insulin-modified IVGTT	Prospective cohort	African American	187 NGT, 101 IGT Total = 288	54.2 ± 0.7	43/57	NGT and IGT	Non-Hispanic white	229	Higher AIR in BA compared to WE when adjusted for age, sex, BMI, and WHR. Greater insulin resistance in BA by Si	9
Haffner et al. [38]	OGTT Insulin-modified IVGTT	Prospective cohort	African American (overweight and obese)	153	57.1 ± 0.7	45/55	T2D	Non-Hispanic white (overweight and obese)	105	OGTT: no difference in fasting and 2 h insulin. AIR higher in BA. No ethnic difference in Si. Adjusted for age, sex, obesity and WHR, fasting glucose, and therapy	9
Osei et al. [34]	OGTT	Cross-sectional	Native Ghanaian, US immigrant Ghanaian, and African American	50, 31, and 66 Total = 147	34.1 ± 1.0, 37.2 ± 1.6, and 33.3 ± 0.9	39/61	NGT	White American	39	Higher peak and iAUC insulin in BA compared to WE, no difference in C-peptide. Basal and postprandial HIE (by C-peptide to insulin molar ratio) lower in BA	9
Chiu et al. [64]	Hyperglycaemic clamp	Cross-sectional	African American	9	26	56/44	NGT	White American	34	Higher second-phase insulin response in BA, no difference in the 1st phase. Lower insulin sensitivity index in BA	7
Melby et al. [49]	Insulin-modified IVGTT	Cross-sectional	African American	9	22.5 ± 0.7	0/100	NGT	White American	8	Age-matched ethnic groups. Higher AIR in BA and lower insulin sensitivity by Si in BA. Similar age, BMI, and percentage body fat between ethnicities	9
Chiu et al. [65]	Hyperglycaemic clamp	Cross-sectional	African American	11	25	45/55	NGT	White American	46	Higher 1st and 2nd phase insulin response in BA, lower insulin sensitivity index	6
Chen et al. [50]	Glucose challenge test (insulin and C-peptide 1-hour post 50 g glucose load)	Prospective cohort	African American pregnant women	343	20.6 ± 0.2	0/100	NGT	White American pregnant women	118	Insulin and insulin to glucose ratio higher, but C-peptide and C-peptide to insulin ratio lower, in BA vs. WE. Similar BMI in both ethnic groups	8
Jensen et al. [40]	OGTT	Cohort	African American	55	41.8 ± 1.9	38/62	NGT, IFG/IGT, and T2D	White American	217	Insulinogenic index adjusted for insulin sensitivity ((*Δ*I30/*Δ*G30)/HOMA-IR) higher in NGT BA vs. WE. No difference in IFG/IGT and T2D	7
Punyadeera et al. [26]	MMTT	Cross-sectional	Native black South African (lean and obese)	8 (lean) and 9 (obese) Total = 17	31.9 ± 3.0 (lean) and 42.5 ± 2.6 (obese)	0/100	NGT	White South African (lean and obese)	17	Higher insulin levels at 30 mins post meal in obese BA compared to obese WE. No difference in total insulin area under the curve between ethnic groups	8
Velasquez-Mieyer et al. [42]	3 h OGTT	Cross-sectional	African American (severely obese)	16	36 ± 2	Not available by ethnicity	NGT and IGT	White American (severely obese)	26	Insulin iAUC and CIR at 30 mins higher in BA vs. WE, no significant difference in iAUC C-peptide. Similar WBISI and fat mass by DXA between ethnic groups	8
Stefan et al. [59]	OGTT, MMTT, and IVGTT	Cross-sectional	African American	30	31 ± 1	66/33	NGT	White American	30	No ethnic difference in AUC insulin post OGTT or MMTT. Higher AIR after IVGTT in BA, when adjusted for insulin sensitivity by M-Low (from 2-step hyperinsulinaemic-euglycaemic clamp). BA and WE matched for age, sex, BMI, WHR, and percentage body fat.	9
Torrens et al. [41]	Fasting glucose and insulin to derive HOMA%B	Prospective cohort	African American	746	46 ± 2.7	0/100	Nondiabetic; included subjects with IFG	Non-Hispanic white	1359	HOMA%B higher in BA after controlling for alcohol consumption, waist circumference, triglycerides, and prevalence of IFG	8
Albu et al. [43]	Insulin- or tolbutamide-modified IVGTT	Cross-sectional	African American	32	36.8 ± 1.3	0/100	NGT and IGT	White American	28	Higher AIR in BA, which persisted when adjusted for insulin sensitivity index and measures of adiposity and skeletal muscle	7
Boule et al. [61]	IVGTT	Pre/postinterventional	African American	173	32.9 (30.8-35.0) (F) 33.1 (30.2-36.0) (M)	37/63	Nondiabetic	White American	423	AIRg and DI higher and Si lower in BA vs WE	8
Reimann et al. [28]	OGTT	Cross-sectional	Black South African (with and without abdominal obesity)	86	27 (23, 30) (without abdominal obesity) 32 (28, 35) (with abdominal obesity)	0/100	Nondiabetic	White South African (with and without abdominal obesity)	90	No ethnic difference in age, BMI, or insulin resistance by HOMA-IR. 2 h C-peptide post glucose significantly higher in BA vs. WE in the group with abdominal obesity. No ethnic difference in post glucose C-peptide in the lean group	7
Elbein et al. [44]	OGTT Insulin- or tolbutamide-modified IVGTT	Case control	African American	159	38.4 ± 9.2	34/66	NGT and IGT	European American	344	OGTT: area under curve insulin and insulinogenic index higher in BA. AIRg and DI higher in BA, no significant difference in Si	9
Herman et al. [35]	OGTT	RCT (data obtained before randomisation)	African American	752	50.5 ± 10.1	26/74	IGT	White American	2117	Higher CIR in BA in the context of greater insulin resistance in BA by HOMA IR and higher BMI	7
Rasouli et al. [60]	OGTT IVGTT Arginine stimulation test in subgroup	Cross-sectional	African American (obese, with and without family hx of T2D)	121	38 (36-39)	41/59	NGT	White American (obese, with and without family hx of T2D)	212	No difference in 2 h OGTT insulin or insulin area under the curve, but higher AIRg in BA. No difference in AIRmax, lower DI max. BA had lower Si but higher disposition index. No difference in age, BMI, or WHR between ethnic groups	9
Goedecke et al. [27]	OGTT Insulin-modified IVGTT	Cross-sectional	Native black South African (lean and obese)	29	24 ± 2 (lean) and 28 ± 1 (obese)	0/100	NGT	White South African (lean and obese)	28	Higher insulinogenic index 30 mins in BA, but no difference when adjusted for insulin sensitivity. Higher AIR and lower Si in BA, adjusted for visceral and subcut adipose volume. No ethnic difference in DI. No ethnic difference in BMI, WHR, or body fat % by DXA	9
Willig et al. [51]	Insulin- or tolbutamide-modified IVGTT	Cross-sectional	African American	87	35.3 ± 4.5	0/100	Nondiabetic	European American	68	AIRg higher and Si lower in BA. No ethnic difference in age and BMI	8
Chandler-Laney et al. [52]	Insulin-modified IVGTT	Cross-sectional	African American	43	25.9 ± 3.4 (premenopausal), 55.7 ± 4.2 (postmenopausal)	0/100	NGT	European American	63	Higher X0 (acute C-peptide secretion), Phi1 and Phi(tot) in BA after adjusting for age. Lower Si in BA, after adjusting for body fat % by DXA	9
Goree et al. [53]	Insulin-modified IVGTT	Cross-sectional	African American	42	Premenopausal 24.8 ± 3.3 Postmenopausal 56.6 ± 5.1	0/100	NGT	European American	64	AIRg and DI higher in BA. AIR remained higher after adjusting for Si.	9
Chow et al. [54]	Insulin-modified IVGTT	Cross-sectional	African American	17	36 ± 9	0/100	Nondiabetic	White American	17	AIRg and DI higher in BA, Si lower. Matched for age and BMI. Lower WHC and higher body fat % in BA	7
Ladson et al. [55]	OGTT	Case control	African American with PCOS	36	27.9 ± 5.0	0/100	Nondiabetic	White American with PCOS	63	Higher fasting insulin and insulinogenic index (30 mins) in BA. Similar BMI and WHR in both ethnic groups	6
Szczepaniak et al. [45]	Insulin-modified IVGTT	Cross-sectional	African American	20	37 ± 3	35/65	NGT and IGT	Non-Hispanic white	30	Higher AIRg and DI in BA, lower Si. Ethnic groups matched for age, sex, BMI, and BP. Adjusted for HbA1c	8
Goff et al. [33]	IVGTT	Cross-sectional	UK Afro-Caribbean	35	42.6 ± 7 (F) 44.9 ± 9.7 (M)	29/71	Nondiabetic	UK white	417	When adjusted for age and BMI: higher AIRg and lower Si in BA. No ethnic difference in DI	7
Ebenibo et al. [62]	IVGTT	Prospective cohort	African American	142	40.2 ± 10.7 (HbA1c < 5.7%), 46.5 ± 8.9 (HbA1c 5.7-6.4%)	25/75	NGT	White American	138	Within each HbA1c group, BA had higher AIRg and DI. Similar insulin sensitivity between ethnic groups by hyperinsulinaemic-euglycaemic clamp	9
Ferguson et al. [58]	HOMA%B Insulin-modified IVGTT	Pre/postinterventional	African American	42	26 (median), 9 (IQR) (F) 27 (median), 18 (IQR) (M)	45/55	Nondiabetic	European American	106	Higher HOMA%B in BA Lower Si and higher AIR and DI in BA, adjusted for age, sex, and BMI	8
Healy et al. [36]	HOMA%B OGTT Insulin-modified IVGTT	Cross-sectional	African American (obese)	84	46.4 ± 10.2	7/93	Prediabetic (IFG and IGT)	White American (obese)	61	HOMA%B lower in BA OGTT fasting and post glucose C-peptide levels lower in BA, no difference in insulinogenic index Higher AIRg in BA (not significant), similar Si, and significantly higher DI in BA. BA had higher BMI and higher body fat % by DXA.	9
Goff et al. [29]	MMTT plus high-fructoseor high-glucose feeding	Cross-sectional	UK black African	9	38.3 ± 2.0	100/0	Nondiabetic	UK white	417	No significant ethnic difference in insulin or C-peptide iAUC post feeding	7
Owei et al. [46]	IVGTT	Prospective cohort	African American (with parental T2D)	184	43.2 ± 10.0	Not reported by ethnicity	NGT and IGT	European American (with parental T2D)	151	Higher AIR in BA. Lower insulin sensitivity by hyperinsulinaemic-euglycaemic clamp in blacks. BA younger and higher BMI	8
Shah et al. [47]	Hyperglycaemic clamp Arginine-stimulated insulin response	Cross-sectional	African American	24	Not reported by ethnicity	Not reported by ethnicity	NGT, IGT, and T2D	White American	74	Higher acute insulin response and DI in BA, late-phase insulin tended to be higher in BA. No ethnic difference in insulin sensitivity index	8
Osei et al. [37]	Fasting parameters to determine HOMA%B OGTT Insulin-modified IVGTT	Cross-sectional	Overweight/obese African American	67	46.3 ± 10.3	0/100	Prediabetic (IFG and IGT)	Overweight/obese white American	28	HOMA%B: no ethnic difference OGTT: fasting C-peptide and peak C-peptide lower in BA, fasting and mean insulin tended to be higher IVGTT: AIR higher in BA, not significant. DI significantly higher in BA. BA had higher BMI and higher percentage body fat by DEXA. Insulin sensitivity by Si same between ethnic groups	9
Piccinini et al. [56]	Insulin-modified IVGTT	Cross-sectional	African American	18	25 ± 4	0/100	NGT	European American	29	Insulin secretion rate (ISR) as modelled by C-peptide higher in BA vs. WE	8
Mohandas et al. [30]	MMTT Hyperglycaemic clamp	Cross-sectional	UK black African	19	54.1 ± 7.7	100/0	T2D (recently diagnosed)	UK white	15	MMTT: fasting and AUC C-peptide lower in BA, no difference in insulin AUC HC: second-phase C-peptide lower in BA, no difference in insulin iAUC. Groups matched for age, BMI, HbA1c, and duration of diabetes	9

AIR: acute insulin response; BA: black African; BMI: body mass index; CIR: corrected insulin response; DI: disposition index; DXA: dual-energy X-ray absorptiometry; FHx: family history; HIE: hepatic insulin extraction; HOMA%B: homeostatic model assessment of beta cell function; HOMA-IR: homeostatic model assessment of insulin resistance; iAUC: incremental area under the curve; IGT: impaired glucose tolerance; IVGTT: intravenous glucose tolerance test; mNOS: modified Newcastle-Ottawa scale; MMTT: mixed meal tolerance test; NGT: normal glucose tolerance; OGTT: oral glucose tolerance test (refers to 2-hour post 75 g oral glucose); PCOS: polycystic ovarian syndrome; RCT: randomised controlled trial; Si: insulin sensitivity index; T2D: type 2 diabetes; WBISI: whole-body insulin sensitivity index; WE: white European; WHR: waist-hip ratio.

**Table 2:** Ethnic comparison of insulin responses.

	Insulin response by ethnicity		
	BA > WE	No significant ethnic difference	BA < WE
Models based on fasting measures (HOMA%B)	[41] (NGT and IFG) [58]	[37] (IFG/IGT)	[32] (T2D) [36] (IFG/IGT)

*Cumulativen(fasting)*	**2253**	**95**	**4709**

Oral nutrient stimulation	[34] [50] [48] [40] (NGT) [42] [35] [55] [44] (NGT and IGT) [28] (in obese only)	[26] (BA > WE at 30 mins, but no difference in total iAUC) [31] [40] (IGT and T2D) [59] [60] [27] [36] (IFG/IGT) [37] (IFG/IGT) [38] (T2D) [30] (T2D) [28] (in lean only) [29]	[24] [25]

*Cumulativen(oral)*	**4541**	**2068**	**33**

IV glucose stimulation	[63] [39] (NGT and IGT) [64] (second-phase response only) [65] [49] [59] [43] (NGT and IGT) [60] [27] [54] [33] [58] [46] (NGT and IGT) [45] (NGT and IGT) [62] [36] (IFG/IGT) [47] (NGT, IGT, and T2D) [37] (IFG/IGT) [38] (T2D) [53] [51] [44] (NGT and IGT) [61]	[30] (T2D)	[25]

*Cumulativen(IV)*	**4461**	**34**	**18**

NGT and nondiabetic subjects, unless otherwise specified. Cumulative *n* fasting, oral, and IV refer to the total number of participants (BA and WE) in the studies using fasting measures, oral nutrient stimulation, and intravenous glucose stimulation techniques, respectively (note that each study may be presented in more than one category). BA: black African; WE: white European; NGT: normal glucose tolerance; IFG: impaired fasting glucose; IGT: impaired glucose tolerance; T2D: type 2 diabetes.

**Table 3:** Ethnic comparison of insulin secretory function as determined by C-peptide levels.

	Insulin response by ethnicity		
	BA > WE	No significant ethnic difference	BA < WE
NGT	[48] (OGTT) [52] (IVGTT) [56] (IVGTT) [28] (OGTT, obese only)	[34] (OGTT) [63] (IVGTT) [28] (OGTT, lean only) [29] (MMTT)	[50] (GCT) [25] (oral glucose, IV tolbutamide and glucagon)

“Prediabetic”			[36] (OGTT) [37] (OGTT)

T2D			[30] (MMTT, HC)

Mixed		[31] (OGTT) [42] (OGTT)	

*Cumulativen*	**262**	**1005**	**753**

Cumulative *n* refers to the total number of participants (BA and WE) in the studies. BA: black African; WE: white European; NGT: normal glucose tolerance; T2D: type 2 diabetes; IVGTT: intravenous glucose tolerance test; OGTT: oral glucose tolerance test; GCT: glucose challenge test; MMTT: mixed meal tolerance test; HC: hyperglycaemic clamp.

## Appendix

### A. Search Strategy for Ovid Medline

1. exp African Continental Ancestry Group/
2. afr∗.mp.
3. ghana∗.mp.
4. nigeria∗.mp.
5. caribbean.mp.
6. beta cell function.mp.
7. insulin secretion.mp.
8. insulin clearance.mp.
9. acute insulin response.mp.
10. beta cell respons∗.mp.
11. insulinogenic index.mp.
12. exp European Continental Ancestry Group/
13. white european.mp.
14. non-hispanic white.mp.
15. caucasian.mp.
16. exp DIABETES MELLITUS/ or exp Adult/
17. diab∗.mp.
18. glucose toleran∗.mp.
19. non-diab∗.mp.
20. healthy.mp.
21. 1 or 2 or 3 or 4 or 5
22. 6 or 7 or 8 or 9 or 10 or 11
23. 12 or 13 or 14 or 15
24. 16 or 17 or 18 or 19 or 20
25. 21 and 22 and 23 and 24

### B. Newcastle-Ottawa Scale, Modified for Cross-Sectional Studies

The most appropriate statements are selected. Studies can score a maximum of 10 stars.

#### B.1. *Selection: Maximum 5 Stars*

1. Representativeness of the sample
  - Truly representative of the average in the target population (all subjects or random sampling)^∗^
  - Somewhat representative of the average in the target population (nonrandom sampling)^∗^
  - No description of the sampling strategy
2. Selected group of users
  - Selection of individuals to exclude factors that will bias results (e.g., medications affecting glucose metabolism)^∗^
  - No relevant/systematic selection
3. Sample size
  - Justified and satisfactory (power calculation included)^∗^
  - Not justified
4. Diagnosis
  - Characterisation of the diagnosis of diabetes subtype^∗∗^
  - Diabetes subtype is provided^∗^
  - No information regarding diabetes subtype

#### B.2. *Comparability: Maximum 2 Stars*

1. The subjects in different outcome groups are comparable, based on the study design or analysis. Confounding factors are controlled:
  - The study controls for the most important factor (BMI)^∗∗^
  - The study controls for any additional factor (e.g., age, sex, insulin sensitivity, and diet)^∗^

#### B.3. *Outcome: Maximum 3 Stars*

1. Ascertainment of the method
  - Validated measurement method (interassay CV included)^∗∗^
  - Nonvalidated measurement method, but the method is available or described^∗^
  - No description of the measurement tool
2. Statistical test
  - The statistical test used to analyse the data is clearly described and appropriate, and the measurement of the association is presented (including SD/SE and the probability level (*p* value))^∗^
  - The statistical test is not appropriate, not described, or incomplete

## Abbreviations

AIRg: Acute insulin response to glucose
BA: Black African(s)
CIR: Corrected insulin response
DXA: Dual-energy X-ray absorptiometry
DI: Disposition index
HIE: Hepatic insulin extraction
HOMA%B: Homeostatic model assessment of beta cell function
HOMA-IR: Homeostatic model assessment of insulin resistance
iAUC: Incremental area under the curve
IFG: Impaired fasting glucose
IGT: Impaired glucose tolerance
IVGTT: Intravenous glucose tolerance test
MMTT: Mixed meal tolerance test
OGTT: Oral glucose tolerance test
Si: Insulin sensitivity index
T2D: Type 2 diabetes
WBISI: Whole-body insulin sensitivity index
WE: White European(s).
